# The Integrin Signaling Network Promotes Axon Regeneration via the Src–Ephexin–RhoA GTPase Signaling Axis

**DOI:** 10.1523/JNEUROSCI.2456-20.2021

**Published:** 2021-06-02

**Authors:** Yoshiki Sakai, Mayuka Tsunekawa, Kohei Ohta, Tatsuhiro Shimizu, Strahil Pastuhov, Hiroshi Hanafusa, Naoki Hisamoto, Kunihiro Matsumoto

**Affiliations:** Division of Biological Science, Graduate School of Science, Nagoya University, Nagoya 464-8602, Japan

**Keywords:** axon regeneration, *C. elegans*, inside-out, integrin, RhoA, Src

## Abstract

Axon regeneration is an evolutionarily conserved process essential for restoring the function of damaged neurons. In *Caenorhabditis elegans* hermaphrodites, initiation of axon regeneration is regulated by the RhoA GTPase–ROCK (Rho-associated coiled-coil kinase)–regulatory nonmuscle myosin light-chain phosphorylation signaling pathway. However, the upstream mechanism that activates the RhoA pathway remains unknown. Here, we show that axon injury activates TLN-1/talin via the cAMP–Epac (exchange protein directly activated by cAMP)–Rap GTPase cascade and that TLN-1 induces multiple downstream events, one of which is integrin inside-out activation, leading to the activation of the RhoA–ROCK signaling pathway. We found that the nonreceptor tyrosine kinase Src, a key mediator of integrin signaling, activates the Rho guanine nucleotide exchange factor EPHX-1/ephexin by phosphorylating the Tyr-568 residue in the autoinhibitory domain. Our results suggest that the *C. elegans* integrin signaling network regulates axon regeneration via the Src–RhoGEF–RhoA axis.

**SIGNIFICANCE STATEMENT** The ability of axons to regenerate after injury is governed by cell-intrinsic regeneration pathways. We have previously demonstrated that the *Caenorhabditis elegans* RhoA GTPase–ROCK (Rho-associated coiled-coil kinase) pathway promotes axon regeneration by inducing MLC-4 phosphorylation. In this study, we found that axon injury activates TLN-1/talin through the cAMP–Epac (exchange protein directly activated by cAMP)–Rap GTPase cascade, leading to integrin inside-out activation, which promotes axonal regeneration by activating the RhoA signaling pathway. In this pathway, SRC-1/Src acts downstream of integrin activation and subsequently activates EPHX-1/ephexin RhoGEF by phosphorylating the Tyr-568 residue in the autoinhibitory domain. Our results suggest that the *C. elegans* integrin signaling network regulates axon regeneration via the Src–RhoGEF–RhoA axis.

## Introduction

The ability of neurons to regenerate damaged axons is essential for functional recovery. The regeneration of axons after injury requires the induction of multiple intracellular changes. To achieve this regeneration, the axon undergoes the following processes: local cytoskeletal reorganization to promote growth cone formation, lipid and protein transport for axon outgrowth, and activation of transcription factors that trigger regenerative programs (He and Jin, 2016). Therefore, manipulation of these processes could be an attractive strategy for therapeutic intervention to improve neuronal regeneration. However, the underlying molecular mechanisms that regulate these processes are not fully understood.

Following nerve injury, the end of the damaged axon is transformed into a growth cone-like structure. This is a crucial step in mounting a successful regenerative response. In particular, the cytoskeletal reorganization that accompanies the generation of the new growth cone is essential for the intrinsic ability to regenerate (Bradke et al., 2012). Growth cone formation and axonal regeneration require a number of changes in actin cytoskeletal dynamics and alterations in microtubule stability (Coles and Bradke, 2015). During axon outgrowth, the Rho family of guanosine triphosphatases (GTPases), such as RhoA, Rac1, and Cdc42, plays an important role in transducing signals that lead to the reorganization of the actin cytoskeleton within the growth cone (Hall, 1998). Rho GTPases are molecular switches that cycle between an inactive GDP-bound form and a GTP-bound active conformation (Boguski and McCormick, 1993). The formation of active Rho GTPases is accelerated by guanine nucleotide exchange factors (GEFs; Cerione and Zheng, 1996). Activated Rho GTPases can interact with effector proteins, which, in turn, trigger a variety of cellular responses. Among RhoA effectors, Rho-associated coiled-coil kinase (ROCK) is known to play a key role in actin organization through myosin activation (Bishop and Hall, 2000).

**Figure 1.**
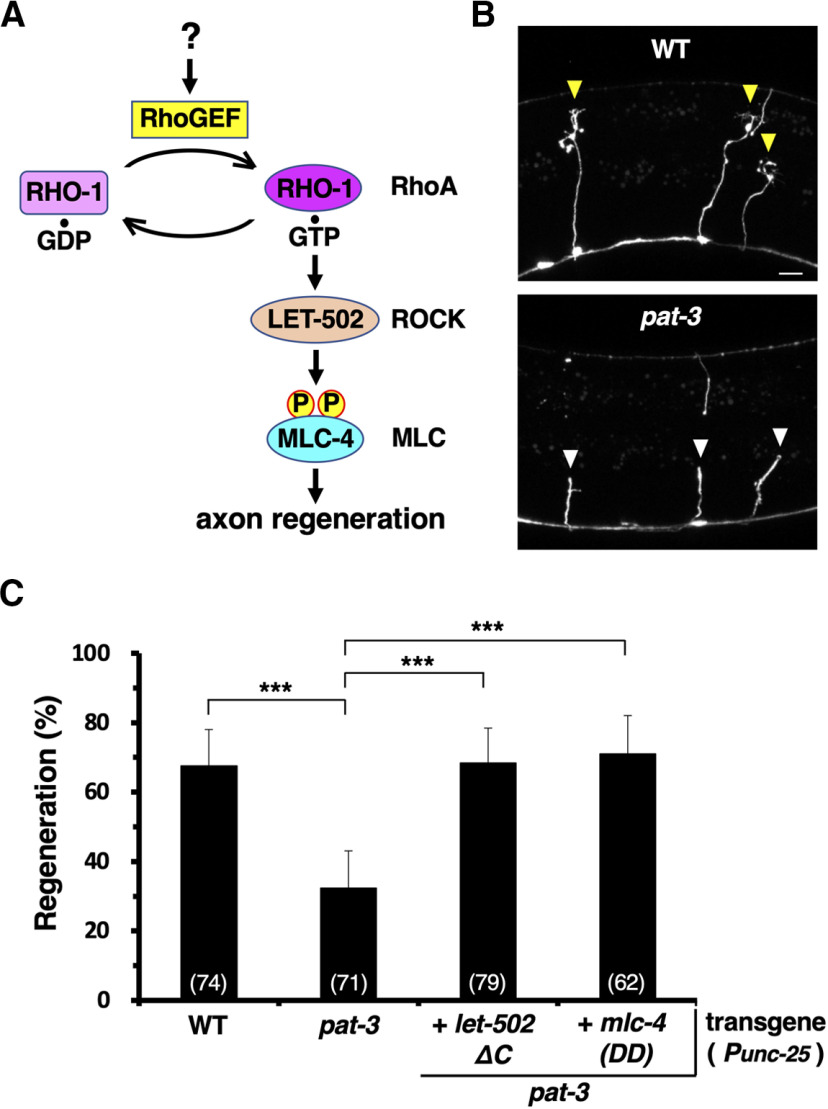
PAT-3 functions in the RHO-1–MLC-4 phosphorylation pathway to regulate axon regeneration. ***A***, The RHO-1/RhoA–LET-502/ROCK–MLC phosphorylation signaling pathway regulating axon regeneration in *C. elegans*. ***B***, Representative D-type motor neurons in wild-type (WT) and *pat-3* mutant animals 24 h after laser surgery. In wild-type animals, severed axons exhibited regenerating growth cones (yellow arrowheads). In *pat-3* mutants, the proximal ends of axons failed to regenerate (white arrowheads). Scale bar, 10 µm. ***C***, Percentages of axons that initiated regeneration 24 h after laser surgery in the young adult stage. The numbers of axons examined are shown. Error bars indicate 95% confidence intervals. ***p < 0.001, as determined by Fisher's exact test.

The nematode *Caenorhabditis elegans* is a valuable model for elucidating the molecular mechanisms involved in axon regeneration (Yanik et al., 2004). Genetic studies in *C. elegans* have identified common biological pathways that use conserved molecules to regulate regeneration (Hammarlund et al., 2009; Nix et al., 2011; Bejjani and Hammarlund, 2012). We have recently reported that the *C. elegans* RhoA homolog RHO-1 promotes axon regeneration of motor neurons by activating the downstream effector LET-502, the *C. elegans* homolog of ROCK (Shimizu et al., 2018). Activated LET-502 phosphorylates nonmuscle myosin light-chain (MLC) MLC-4. Phosphorylation of MLC activates the Mg^2+^-dependent ATPase activity of nonmuscle myosin II, resulting in actin–myosin interaction (Amano et al., 1996). Thus, the RHO-1/RhoA–LET-502/ROCK pathway positively regulates *C. elegans* axon regeneration through MLC-4 phosphorylation (Fig. 1*A*). However, it is still unknown how the RHO-1 pathway is activated on axon injury.

Integrin signaling is one well characterized pathway that regulates the activation status of Rho GTPases (Huveneers and Danen, 2009). Integrins are α–β-heterodimeric transmembrane receptors that mediate cell–extracellular matrix (ECM) interactions (Hynes, 1987). Upon binding to ECM proteins, integrins can transduce signals that activate Rho GTPases, thereby initiating cytoskeletal rearrangement. Integrin function is regulated by inside-out and outside-in signaling (Kim et al., 2011). Inside-out signaling converts the extracellular domain into a high-affinity receptor, allowing interaction with a ligand, which alters the activity of intracellular components such as protein kinases and GTPases (outside-in signaling). Thus, integrins are bidirectional signaling receptors that transmit information into and out of cells. A critical step in the activation of inside-out signaling is the binding of the cytoskeletal protein talin to the cytoplasmic domain of β-integrin. This interaction leads to the dissociation of the integrin α- and β-transmembrane domains that modulate downstream signaling (Kim et al., 2003).

In the mammalian nervous system, integrins are involved in axon growth, synaptogenesis, and axon regeneration (Nikonenko et al., 2003; Eva and Fawcett, 2014). The *C. elegans* integrins INA-1/integrin α and PAT-3/integrin β also participate in axon regeneration (Pastuhov et al., 2016; Hisamoto et al., 2019). In this study, we investigated the relationship between integrin signaling and RhoA activation in the regulation of axon regeneration in *C. elegans*. We found that TLN-1/talin-mediated integrin inside-out activation promotes axon regeneration by activating the RHO-1/RhoA signaling pathway. We showed that nonreceptor tyrosine kinase Src, an important effector downstream of integrin, phosphorylates EPHX-1/ephexin RhoGEF at the Tyr-568 residue located in the autoinhibitory region. This phosphorylation relieves the autoinhibitory regulation of EPHX-1, leading to its activation. These results suggest that the integrin–Src–RhoGEF cascade activates the RhoA–ROCK pathway in axon regeneration.

## Materials and Methods

### 

#### 

##### C. elegans strains.

The *C. elegans* strains used in this study are listed in Table 1. All strains were maintained on nematode growth medium plates and fed with bacteria of the OP50 strain by the standard method, as described previously (Brenner, 1974).

**Table 1.** Strains used in this study

Strain	Genotype
KU501	*juIs76 II*
KU1265	*juIs76 II; pat-3(gk804163) III*
KU1356	*juIs76 II; pat-3(gk804163) III; kmEx1405 [Punc-25::let-502*Δ*C]*
KU1357	*juIs76 II; pat-3(gk804163) III; kmEx1406 [Punc-25::venus::mlc-4(DD)]*
KU1358	*tln-1(e259) I; juIs76 II*
KU1359	*tln-1(e259) I; juIs76 II; pat-3(gk804163) III*
KU1360	*tln-1(e259) I; juIs76 II; kmEx1405 [Punc-25::let-502*Δ*C]*
KU1361	*tln-1(e259) I; juIs76 II; kmEx1406 [Punc-25::venus::mlc-4(DD)]*
KU1362	*juIs76 II; pat-3(D768R) III*
KU1363	*tln-1(e259) I; juIs76 II; pat-3(D768R) III*
KU1364	*juIs76 II; ina-1(R1114D) III*
KU1365	*tln-1(e259) I; juIs76 II; ina-1(R1114D) III*
KU1366	*juIs76 II; pat-3(gk804163; D768R) III*
KU1367	*juIs76 II; pat-3(gk804163) ina-1(R1114D) III*
KU1368	*juIs76 II; rap-1(pk2082) IV*
KU1369	*juIs76 II; rap-2(gk11) V*
KU1370	*tln-1(e259) I; juIs76 II; rap-2(gk11) V*
KU1371	*tln-1(L347A) I; juIs76 II; rap-2(gk11) V*
KU1372	*tln-1(L347A::CAAX) I; juIs76 II; rap-2(gk11) V*
KU1373	*juIs76 II; pat-3(D768R) III; rap-2(gk11) V*
KU1374	*juIs76 II; rap-2(gk11) V; kmEx1405 [Punc-25::let-502*Δ*C]*
KU1375	*juIs76 II; deb-1(gk329549) IV*
KU1376	*juIs76 II; deb-1(gk329549) IV; kmEx1405 [Punc-25::let-502*Δ*C]*
KU1377	*juIs76 II; deb-1(gk329549) IV; kmEx1406 [Punc-25::venus::mlc-4(DD)]*
KU1378	*juIs76 II; epac-1(tm3203) III*
KU1379	*juIs76 II; epac-1(tm3203) III; rap-2(gk11) V*
KU1380	*juIs76 II; epac-1(tm3203) III; kmEx1380 [Punc-25::rap-2(G12V)]*
KU1381	*juIs76 II; epac-1(tm3203) III; kmEx1381 [Punc-25::rap-2(S17A)]*
KU1382	*juIs76 II; epac-1(G84E; G440D) III* line #1
KU1383	*juIs76 II; epac-1(G84E; G440D) III* line #2
KU1384	*ephx-1(Y568F) juIs76 II*
KU1385	*ephx-1(Y568E) juIs76 II*
KU1386	*ephx-1(Y568F) juIs76 II; kmEx1405 [Punc-25::let-502*Δ*C]*
KU1387	*juIs76 II; kmEx1387 [Punc-25::ephx-1*Δ*N]*
KU722	*src-1(cj293)/hT2[bli-4(e937) let-?(q782) qIs48] (I;III); juIs76 II*
KU1388	*src-1(cj293)/hT2[bli-4(e937) let-?(q782) qIs48] (I;III); juIs76 II; kmEx1387 [Punc-25::ephx-1*Δ*N]*

##### Plasmids.

*Punc-25::let-502*Δ*C* and *Punc-25::venus::mlc-4(DD)* plasmids were described previously (Shimizu et al., 2018). *Punc-25::rap-2(G12V)* and *Punc-25::rap-2(S17A)* plasmids were generated by inserting the *rap-2* cDNA isolated from a cDNA library into the pSC325 vector, followed by oligonucleotide-directed inverse PCR. The FLAG-RAP-2(G12V or S17A) plasmid was generated by inserting *rap-2(G12V)* or *rap-2(S17A)* cDNA into the pCMV-FLAG vector. The *Punc-25::ephx-1*Δ*N* plasmid was generated by inserting the *ephx-1* cDNA (isoform a) isolated from a cDNA library into the pSC325 vector, followed by oligonucleotide-directed inverse PCR. GFP-EPHX-1(557–656) and GFP-TLN-1(1–431) plasmids were generated by inserting *ephx-1(557–656)* and *tln-1(1–431)* partial cDNAs, respectively, into a pEGFP-C1 vector. The GFP-EPHX-1(557–656; Y568F) plasmid was generated by oligonucleotide-directed inverse PCR using GFP-EPHX-1(557–656) as a template. The pGBD-RHO-1 plasmid was generated by inserting the *rho*−*1* cDNA that lacks the C-terminal CAAX box sequence into the pGBDU vector. pAD-EPHX-1 and pAD-EPHX-1ΔN plasmids were generated by inserting each of the corresponding cDNAs into the pACTII vector. The *Pmyo-2::dsred-monomer* plasmid was described previously (Li et al., 2012).

##### Generation of mutants using CRISPR–Cas9.

*pat-3(D768R)*, *ina-1(R1114D)*, *tln-1(L347A)*, *tln-1(L347A)::CAAX*, *epac-1(G84E; G440D)*, and *ephx-1(Y568F* or *Y568E)* alleles were generated using the CRISPR (clustered regularly interspaced short palindromic repeats)–Cas9 system, as described previously (Dokshin et al., 2018). CRISPR guide RNAs [5′-GUUAUUGAAAGUAGCGUAUU-3′ for *pat-3(D768R)*, 5′-UUUCUUCAAACGAAAUCGUU-3′ for *ina-1(R1114D)*, 5′-AGUAACGUUCUUUGUGGUGA-3′ for *tln-1(L347A)*, 5′-CACUUGAAAACAUUACGAUG-3′ for *epac-1(G84E)*, 5′-CUGCGAGAAGGUGAUGAUUU-3′ for *epac-1(G440D)*, and 5′-AGCAUAUAAUGUUGAUACAA-3′ for *ephx-1(Y568F* or *Y568E)*] and their corresponding 70 nt single-stranded donor template DNAs were synthesized [Integrated DNA Technologies (IDT)], and coinjected with the transactivating CRISPR RNA (IDT), *Streptococcus* pyogenes Cas9 3NLS (IDT) protein, and pRF4(rol-6d) plasmid into KU501 [for *pat-3(D768R)*, *tln-1(L347A), epac-1(G84E; G440D)*, and *ephx-1(Y568F* or *Y568E)*], KU1265 [for *pat-3(gk804163; D768R)* and *pat-3(gk804163) ina-1(R1114D)*], and KU1358 [for *tln-1(e259); ina-1(R1114D)*] strains. The *tln-1(L347A)::CAAX* allele was created by C-terminal tagging of the *tln-1(L347A)* allele with the RAP-2 C-terminal region (MNYVQNKSRQSKSCCSLM) containing the CAAX box motif using the CRISPR guide RNA (5′-UUUCUUCAAACGAAAUCGUU-3′) and the corresponding 200 nt single-stranded donor template DNA. Each F1 animal carrying the transgene was transferred onto a new dish and single-worm PCR was performed, followed by DNA sequencing to detect mutations. The other strains were generated by standard crosses.

##### Transgenic animals.

Transgenic animals were obtained using the standard *C. elegans* microinjection method (Mello et al., 1991). *Pmyo-2::dsred-monomer*, *Punc-25::rap-2(G12V)*, *Punc-25::rap-2(S17A)*, and *Punc-25::ephx-1*Δ*N* plasmids were used in *kmEx1380* [*Punc-25::rap-2(G12V)* (25 ng/µl) + *Pmyo-2::dsred-monomer* (5 ng/µl)], *kmEx1381* [*Punc-25::rap-2(S17A)* (25 ng/µl) + *Pmyo-2::dsred-monomer* (5 ng/µl)], and *kmEx1387* [*Punc-25::ephx-1*Δ*N* (25 ng/µl) + *Pmyo-2::dsred-monomer* (5 ng/µl)], respectively. The *kmEx1405* and *kmEx1406* extrachromosomal arrays have been described previously (Shimizu et al., 2018).

##### Microscopy.

Fluorescent images of transgenic animals were observed under the 100× objective lens of a fluorescence microscope (model ECLIPSE E800, Nikon) and photographed using a CCD camera (Zyla, Oxford Instruments).

##### Axotomy.

Axotomies were performed as described previously (Li et al., 2012). Animals were subjected to axotomy at the young adult stage. Commissures that displayed growth cones or small branches present on the proximal fragment were counted as “regenerated.” Proximal fragments that showed no change after 24 h were counted as “no regeneration.” A minimum of 20 individuals with 1–3 one to three axotomized commissures was observed for most experiments.

##### Biochemical analysis.

Transfection of transgenes into COS-7 cells, preparation of the cell lysates, immunoprecipitation, and immunoblotting using anti-FLAG and anti-GFP antibodies has been described previously (Li et al., 2012).

##### In vitro kinase assays.

GFP-EPHX-1(557–656) and GFP-EPHX-1(557–656; Y568F) proteins were expressed in COS7 cells and immunopurified with an anti-GFP antibody (mouse; stock #M048-3, MBL). Kinase reactions were performed in a final volume of 20 µl buffer consisting of 5 mm MOPS [3-(*N*-morpholino)propanesulfonic acid], pH 7.2, 2.5 mm β-glycerol-phosphate, 5 mm MgCl_2_, 1 mm EGTA, 0.5 mm EDTA, 5 µCi of [γ-^32^P]ATP, 100 μm ATP, and 0.4 µg of recombinant Src (Carna Biosciences). Samples were incubated for 20 min at 30°C, and reactions were terminated by the addition of Laemmli sample buffer and boiling. Samples were resolved by SDS-PAGE and analyzed by autoradiography.

##### Yeast two-hybrid assays.

For yeast two-hybrid analysis, GAL4 AD-EPHX-1 or GAL4 AD-EPHX-1ΔN was cotransformed with either GAL4 DBD-RHO-1 or empty pGBDU vectors into the *Saccharomyces cerevisiae* reporter strain PJ69-4A (*MAT***a** *trp1-901 ura3-52 leu2-3112 his3-200 gal4*Δ *gal80*Δ *Met2::GAL7-lacZ LYS2::GAL1-HIS3 Ade2::GAL2-ADE2*), and yeasts were allowed to grow on SC-Ura-Leu plates. Transformants grown on these plates were then streaked out onto SC-Ura-Leu-His plates with 10 mm 5-aminotriazole and incubated at 30°C for 4 d.

##### Statistical analysis.

Statistical analyses were performed as described previously (Li et al., 2012). Briefly, confidence intervals (95%) were calculated using the modified Wald method and two-tailed *p* values were calculated using Fisher's exact test (https://www.graphpad.com/quickcalcs/contingency1/).

## Results

### PAT-3/integrin β functions in the RhoA–ROCK–MLC phosphorylation pathway to regulate axon regeneration

*C. elegans* axon regeneration after axon injury is regulated by the RHO-1/RhoA–LET-502/ROCK–MLC-4 phosphorylation pathway (Fig. 1*A*; Shimizu et al., 2018). We sought to determine whether integrin functions upstream of the RHO-1 pathway in axon regeneration. We have previously reported that PAT-3/integrin β is involved in axonal regeneration (Hisamoto et al., 2019). Here, we first confirmed the *pat-3* mutant phenotype. We subjected GABA-releasing D-type motor neurons (D neurons) to laser axotomy and subsequently monitored the regrowth of their axons. D neurons extend axons from the ventral to dorsal side of the animal body (Fig. 1*B*). In young adult wild-type animals, ∼70% of severed axons formed growth cones and initiated regeneration within 24 h after injury (Fig. 1*B*,*C*, Table 2). However, in *pat-3(gk804163)* mutants, the frequency of axon regeneration was substantially reduced (Fig. 1*B*,*C*, Table 2).

**Table 2.** Raw data for genotypes tested by axotomy

Strain	Genotype (*juIs76* background)	Axons, *n*	Regenerations, *n* (% of total)	*p* Value	Compared with
KU501*^a^*	Wild type	74	50 (68%)		
KU1265*^a^*	*pat-3(gk804163)*	71	23 (32%)	<0.0001	KU501*^a^*
KU1356	*pat-3(gk804163); kmEx1405 [Punc-25::let-502*Δ*C]*	79	54 (68%)	<0.0001	KU1265*^a^*
KU1357	*pat-3(gk804163); kmEx1406 [Punc-25::venus::mlc-4(DD)]*	62	44 (71%)	<0.0001	KU1265*^a^*
KU501*^b^*	Wild type	55	40 (73%)		
KU1358*^a^*	*tln-1(e259)*	68	23 (34%)	<0.0001	KU501*^b^*
KU1265*^b^*	*pat-3(gk804163)*	47	17 (36%)		
KU1359	*tln-1(e259); pat-3(gk804163)*	51	15 (29%)	0.6927, 0.5226	KU1358*^a^*, KU1265*^b^*
KU1360	*tln-1(e259); kmEx1405 [Punc-25::let-502*Δ*C]*	67	42 (63%)	0.0010	KU1358*^a^*
KU1361	*tln-1(e259); kmEx1406 [Punc-25::venus::mlc-4(DD)]*	49	33 (67%)	0.0004	KU1358*^a^*
KU501*^c^*	Wild type	60	37 (62%)		
KU1358*^b^*	*tln-1(e259)*	46	13 (28%)	0.0008	KU501*^c^*
KU1362	*pat-3(D768R)*	44	26 (59%)	0.8406	KU501*^c^*
KU1363	*tln-1(e259); pat-3(D768R)*	62	38 (61%)	0.0009	KU1358*^b^*
KU501*^d^*	Wild type	37	24 (65%)		
KU1364	*ina-1(R1114D)*	60	38 (63%)	1.0000	KU501*^d^*
KU1358*^c^*	*tln-1(e259)*	54	17 (31%)		
KU1365	*tln-1(e259); ina-1(R1114D)*	60	36 (60%)	0.0027	KU1358*^c^*
KU1265*^c^*	*pat-3(gk804163)*	46	17 (37%)		
KU1366	*pat-3(gk804163; D768R)*	56	36 (64%)	0.0093	KU1265*^c^*
KU1367	*pat-3(gk804163) ina-1(R1114D)*	64	41 (64%)	0.0067	KU1265*^c^*
KU501*^e^*	Wild type	38	24 (63%)		
KU1368	*rap-1(pk2082)*	26	19 (73%)	0.4329	KU501*^e^*
KU1369*^a^*	*rap-2(gk11)*	62	25 (40%)	0.0389	KU501*^e^*
KU1358*^d^*	*tln-1(e259)*	40	12 (30%)		
KU1370	*tln-1(e259); rap-2(gk11)*	49	14 (29%)	0.2327, 1.0000	KU1369*^a^*, KU1358*^d^*
KU1369*^b^*	*rap-2(gk11)*	42	14 (33%)		
KU1371	*tln-1(L347A); rap-2(gk11)*	49	19 (39%)	0.6645	KU1369*^b^*
KU1372	*tln-1(L347A::CAAX); rap-2(gk11)*	78	48 (62%)	0.0041	KU1369*^b^*
KU1369*^c^*	*rap-2(gk11)*	29	10 (34%)		
KU1373	*pat-3(D768R); rap-2(gk11)*	60	21 (35%)	1.0000	KU1369*^c^*
KU1374	*rap-2(gk11); kmEx1405 [Punc-25::let-502*Δ*C]*	55	25 (45%)	0.3618	KU1369*^c^*
KU1375	*deb-1(gk329549)*	58	21 (36%)	0.0004	KU501*^a^*
KU1376	*deb-1(gk329549); kmEx1405 [Punc-25::let-502*Δ*C]*	66	23 (35%)	1.0000	KU1375
KU1377	*deb-1(gk329549); kmEx1406 [Punc-25::venus::mlc-4(DD)]*	51	21 (41%)	0.6940	KU1375
KU501*^f^*	Wild type	46	30 (65%)		
KU1378	*epac-1(tm3203)*	50	19 (38%)	0.0088	KU501*^f^*
KU1369*^d^*	*rap-2(gk11)*	46	16 (35%)	0.0064	KU501*^f^*
KU1379	*epac-1(tm3203); rap-2(gk11)*	45	19 (42%)	0.6817, 0.5219	KU1378, KU1369*^d^*
KU1380	*epac-1(tm3203); kmEx1380 [Punc-25::rap-2(G12V)]*	59	48 (81%)	<0.0001	KU1378
KU1381	*epac-1(tm3203); kmEx1381 [Punc-25::rap-2(S17A)]*	46	24 (52%)	0.2180	KU1378
KU501*^g^*	Wild type	62	41 (66%)		
KU1382	*epac-1(G84E; G440D)* line #1	59	25 (42%)	0.0108	KU501*^g^*
KU1383	*epac-1(G84E; G440D)* line #2	72	29 (40%)	0.0033	KU501*^g^*
KU501*^h^*	wild type	41	28 (68%)		
KU1384	*ephx-1(Y568F)*	49	19 (39%)	0.0063	KU501*^h^*
KU1386	*ephx-1(Y568F); kmEx1405 [Punc-25::let-502*Δ*C]*	56	42 (75%)	0.0003	KU1384
KU1385	*ephx-1(Y568E)*	36	13 (36%)	0.0062	KU501*^h^*
KU501*^i^*	Wild type	50	36 (72%)		
KU1387	*kmEx1387 [Punc-25::ephx-1*Δ*N]*	26	21 (81%)	0.5776	KU501*^i^*
KU722	*src-1(cj293)*	40	9 (23%)	<0.0001	KU501*^i^*
KU1388	*src-1(cj293); kmEx1387 [Punc-25::ephx-1*Δ*N]*	27	15 (56%)	0.0090	KU722

a to i: different controls of the same strain.

Next, we investigated the relationship between PAT-3 and the RHO-1 pathway. The activation of the RHO-1 pathway is bypassed by the introduction of constitutively active downstream effectors. The kinase activity of LET-502/ROCK is usually autoinhibited by its C-terminal region, and truncation of the C-terminus constitutively activates LET-502 kinase activity (Shimizu et al., 2018). We found that the expression of the LET-502 C-terminal truncated mutant (LET-502ΔC) from the *unc-25* promoter in D neurons could suppress the regeneration defect of *pat-3(gk804163)* mutants (Fig. 1*C*, Table 2). Overexpression of LET-502ΔC does not promote axon regeneration in wild-type animals (Shimizu et al., 2018). These results suggest that PAT-3 functions upstream of LET-502 in axon regeneration.

RhoA activates myosin II via ROCK, which phosphorylates Thr-18 and Ser-19 of the MLC protein (Amano et al., 1996). Next, we examined whether PAT-3 signaling promotes axon regeneration by inducing the phosphorylation of MLC-4. In *C. elegans*, MLC-4 is phosphorylated by LET-502 at two highly conserved residues, Thr-17 and Ser-18 (Shimizu et al., 2018). We introduced constitutive phosphomimetic mutations, *T17D* and *S18D*, into the *mlc-4* gene [*mlc-4(DD)*] and found that the expression of MLC-4(DD) could suppress the axon regeneration defect observed in *pat-3(gk804163)* mutants (Fig. 1*C*, Table 2). Expression of MLC-4(DD) in wild-type animals has no effect on the frequency of axon regeneration (Shimizu et al., 2018). Together, these results suggest that PAT-3 functions as an upstream component in the RHO-1–LET-502–MLC-4 phosphorylation pathway to promote axon regeneration.

### TLN-1/talin promotes axon regeneration by inducing integrin inside-out activation

**Figure 2.**
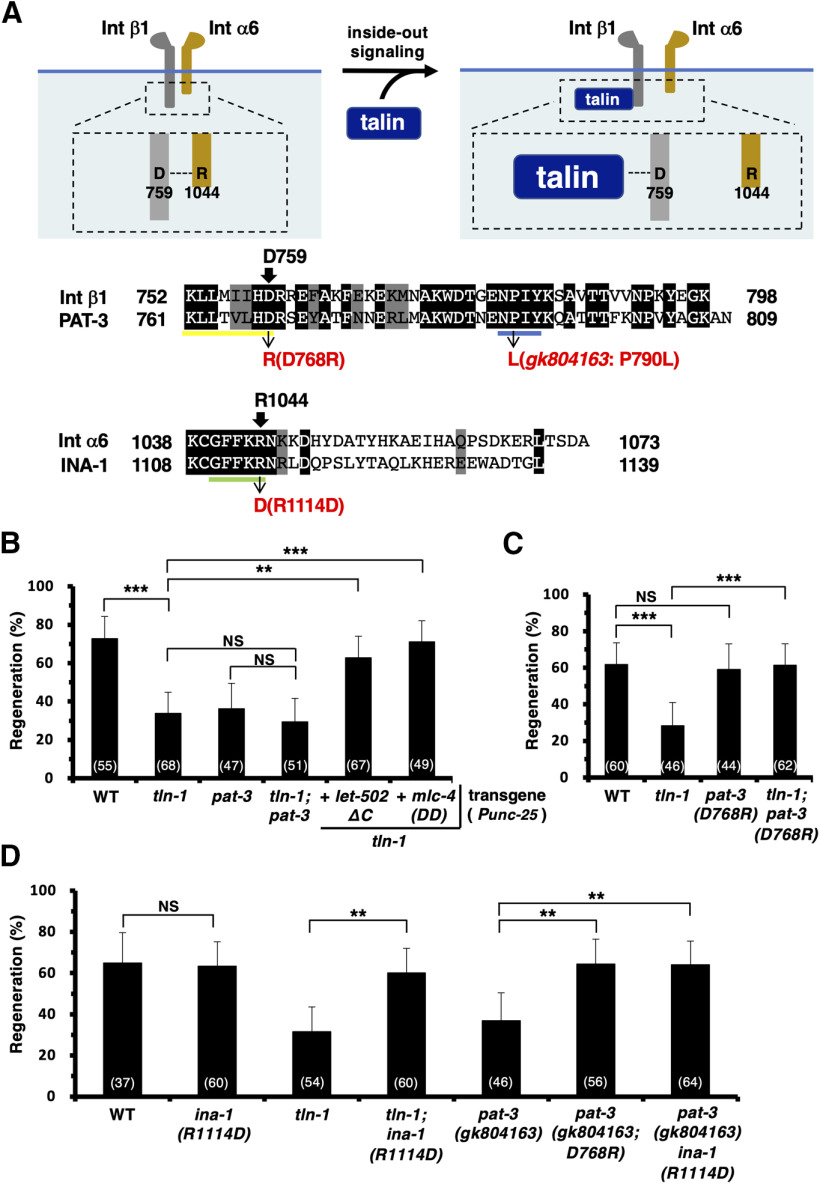
TLN-1 promotes axon regeneration by inducing integrin inside-out activation. ***A***, Talin-mediated integrin inside-out activation. Talin forms a salt bridge with Asp-759 in β1, which disrupts the interaction between β1 Asp-759 and α6 Arg-1044. Sequence alignments of cytoplasmic tails between integrin β1 and PAT-3 and integrin α6 and INA-1 are shown. Identical and similar residues are highlighted with black and gray shading, respectively. The conserved KLLtVLHD, GFFKR, and NPxY motifs are underlined in yellow, green, and blue, respectively. Asp-759 (in β1), Asp-768 (in PAT-3), Arg-1044 (in α6), Arg-1114 (in INA-1), and Pro-790 (in PAT-3) are indicated by arrows. The membranes are shown in blue. ***B*−*D***, Percentages of axons that initiated regeneration 24 h after laser surgery in the young adult stage. The numbers of axons examined are shown. Error bars indicate 95% confidence intervals. **p < 0.01, ***p < 0.001, as determined by Fisher's exact test. NS, Not significant; WT, wild type; Int, integrin.

Integrin function is regulated by inside-out signaling, which is controlled by talin (Fig. 2*A*; Kim et al., 2011). We therefore examined whether *C. elegans* TLN-1/talin is also required for axon regeneration. We found that the frequency of axon regeneration in D neurons was significantly reduced in *tln-1(e259)* mutants (Fig. 2*B*, Table 2). Furthermore, the regeneration defect of *tln-1 (e259); pat-3(gk804163)* double mutants was no greater than that of *tln-1(e259)* or *pat-3(gk804163)* single mutants (Fig. 2*B*, Table 2), suggesting that TLN-1 acts in the same functional pathway as PAT-3. Consistent with this, we found that, similar to *pat-3(gk804163)* mutants, the *tln-1* defect in axon regeneration was suppressed by the expression of LET-502ΔC or MLC-4(DD) from the *unc-25* promoter in D-type motor neurons (Fig. 2*B*, Table 2). Thus, it is likely that PAT-3 and TLN-1 function in the RHO-1–LET-502–MLC-4 phosphorylation pathway to control axon regeneration.

In the *pat-3(gk804163*) mutant, the Pro-790 residue in the membrane-proximal NPxY motif Asn-Pro (790)-Ile-Tyr is replaced with a leucine residue (Fig. 2*A*; Hisamoto et al., 2019). Integrin activation requires this NPxY motif in the β-tail to interact with the PTB (phosphotyrosine-binding) domain in the N-terminal portion of talin (Calderwood et al., 2003). Therefore, the *pat-3(gk804163)* mutant is expected to be defective in integrin activation by talin binding. Functional integrin receptors are heterodimers composed of one α-subunit and one β-subunit. Before integrin activation, they form a salt bridge between Arg (Arg-1044 in integrin α6) in the GFFKR motif and Asp (Asp-759 in integrin β1) in the KLLxIIHD motif, which stabilizes the inactive form of integrin (Fig. 2*A*; Hughes et al., 1996). During integrin inside-out activation, talin binds to the cytoplasmic β-tail and forms a salt bridge with the conserved Asp residue, which disrupts the inhibitory interaction of the β-tail with the conserved Arg residue in α-integrin (Wegener et al., 2007). Therefore, the charge-reversal mutations *D759R* in integrin β1 and *R1044D* in integrin α6, wherein Asp-759 and Arg-1044 are replaced by arginine and aspartic acid residues, respectively, lead to a constitutively active integrin via the disruption of the α–β salt bridge (Fig. 2*A*; Hughes et al., 1996; Laursen et al., 2011). Because the conserved motifs GFFKR and KLLtVLHD are also present in INA-1/integrin α (Arg-1114) and PAT-3/integrin β (Asp-768), respectively (Fig. 2*A*), we predicted that *ina-1(R1114D)* and *pat-3(D768R)* mutations would result in constitutively active integrins. To test this possibility, we generated *ina-1(R1114D)* and *pat-3(D768R)* mutants in the endogenous *ina-1* and *pat-3* loci, respectively, by CRISPR/Cas9 mutagenesis. We found that the *ina-1(R1114D)* and *pat-3(D768R)* mutations were able to suppress the *tln-1(e259)* phenotype of defective axon regeneration (Fig. 2*C*, Table 2). These results support the possibility that TLN-1 promotes axon regeneration through integrin inside-out activation.

The *gk804163* allele of *pat-3* fails to bind to TLN-1 and consequently associates constitutively with INA-1. Thus, the *pat-3(gk804163)* mutation is predicted to have a dominant-negative effect on axon regeneration rather than act as a loss-of-function mutation. Consistent with this prediction, we found that the introduction of the *D768R* mutation into the *pat-3(gk804163)* background could suppress the regeneration defect (Fig. 2*D*, Table 2). Moreover, the *ina-1(R1114D)* mutation was able to suppress the *pat-3(gk804163)* phenotype (Fig. 2*D*, Table 2). These results suggest that the *pat-3(gk804163)* mutation inhibits axon regeneration by constitutively binding to INA-1.

### The RAP-2 GTPase−TLN-1 pathway mediates integrin inside-out activation during axon regeneration

**Figure 3.**
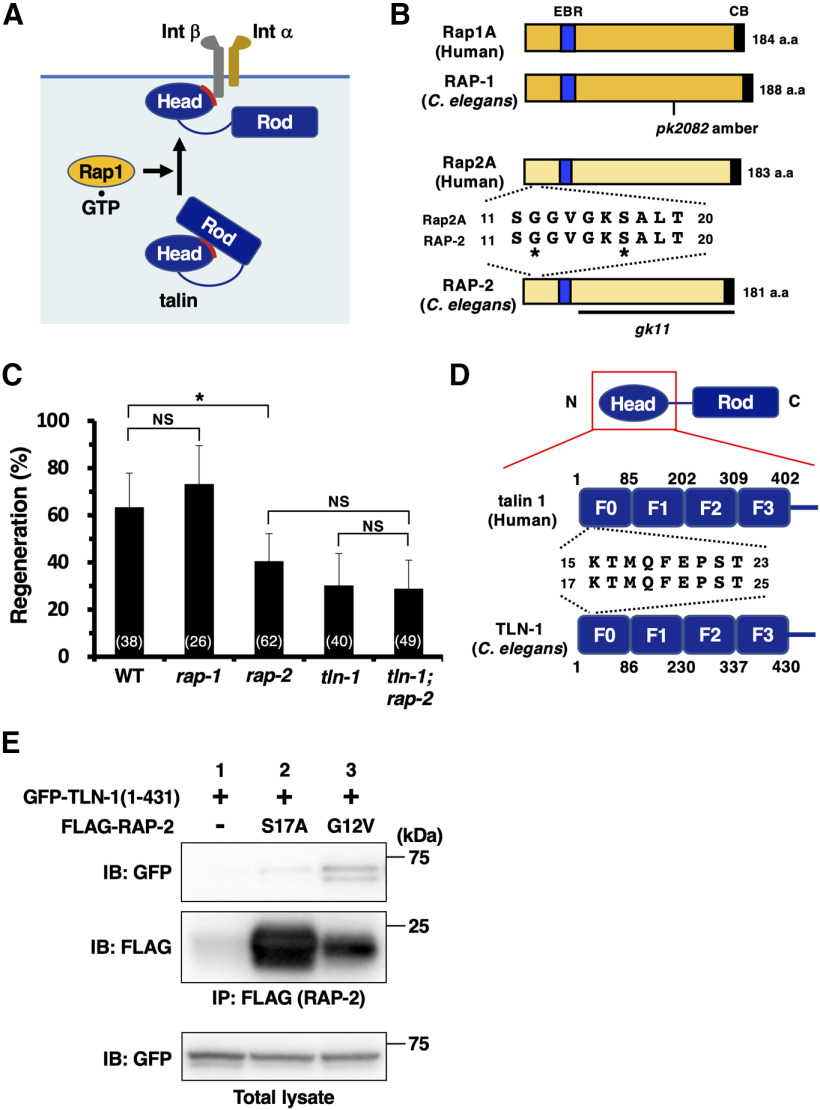
RAP-2 promotes axon regeneration by interacting with TLN-1. ***A***, The Rap1−talin pathway. GTP-bound Rap1 interacts with and recruits talin to the membrane where it is activated, which can expose the integrin β tail binding site of talin. ***B***, Structures of RAP-1 and RAP-2. Schematic diagrams of RAP-1, RAP-2, and their human counterparts, Rap1A and Rap2A, are shown. The effector binding region (EBR) is shown in blue, and the CAAX box (CB) is shown in black. Constitutively active RAP-2(G12V) and inactive RAP-2(S17A) mutations are denoted by asterisks. The *pk2082* allele of *rap-1* is an amber mutation that generates RAP-1(1–129). Bold line underneath the RAP-2 diagram denotes the extent of the deleted region in the *gk11* mutant. ***C***, Percentages of axons that initiated regeneration 24 h after laser surgery in the young adult stage. The numbers of axons examined are shown. Error bars indicate 95% confidence intervals. *p < 0.05, as determined by Fisher's exact test. NS, Not significant. ***D***, Structure of the talin head domain. Schematic diagrams of head domains of talin 1 and TLN-1 are shown. The talin head domain is composed of four subdomains: F0, F1, F2, and F3. ***E***, Interaction of TLN-1 with RAP-2. COS-7 cells were cotransfected with GFP-TLN-1(1 − 431), and FLAG-GTP-bound RAP-2(G12V) or GDP-bound RAP-2(S17A) as indicated. Complex formation was detected by immunoprecipitation (IP) with anti-FLAG antibody, followed by immunoblotting (IB) with anti-GFP antibody. Total lysates were immunoblotted with anti-GFP antibody. WT, Wild type; Int, integrin.

What is the mechanism that triggers activation of talin during axon regeneration? Talin is autoinhibited in the cytosol by the interaction of the N-terminal head domain with the C-terminal rod domain, which prevents the interaction of the head domain with the membrane surface and β-integrin cytoplasmic tail (Goksoy et al., 2008). Autoinhibited talin is recruited to the membrane via its freely accessible head domain in a Rap1 GTPase-dependent manner (Zhu et al., 2017). Therefore, Rap1 is essential for integrin inside-out signaling (Fig. 3*A*). We next examined whether *C. elegans* RAP is also involved in axon regeneration regulated by talin-mediated integrin inside-out activation. The *C. elegans* genome contains the *rap-1* and *rap-2* genes, which encode the mammalian Rap1 and Rap2 homologs, respectively (Fig. 3*B*). We found that *rap-2(gk11)* mutants were defective in axon regeneration, whereas the *rap-1(pk2082)* mutation had no effect on regeneration (Fig. 3*C*, Table 2). The regeneration defect observed in *rap-2(gk11)* mutants was not enhanced by the *tln-1(e259)* mutation (Fig. 3*C*, Table 2), suggesting that RAP-2 and TLN-1 act in the same functional pathway.

Several studies have defined a region in talin that binds directly to Rap1 (Zhu et al., 2017; Gingras et al., 2019). The talin head domain is composed of four subdomains, F0, F1, F2, and F3, and the interaction site with Rap1 lies in the F0 domain (Fig. 3*D*). This region is well conserved between *C. elegans* TLN-1 and mammalian talin (Fig. 3*D*). We examined whether RAP-2 binds to the TLN-1 head domain (amino acids 1−431) in a GTP binding-dependent manner. GFP-tagged TLN-1(1−431) was cotransfected into mammalian COS-7 cells with FLAG-tagged GTP-bound RAP-2(G12V) or GDP-bound RAP-2(S17A). Coimmunoprecipitation experiments revealed that RAP-2(G12V), but not RAP-2(S17A), interacted with TLN-1(1 − 431) (Fig. 3*E*). These results suggest that direct binding of TLN-1 to RAP-2 via the TLN-1 head domain is evolutionarily conserved and dependent on the GTP-binding form of RAP-2.

**Figure 4.**
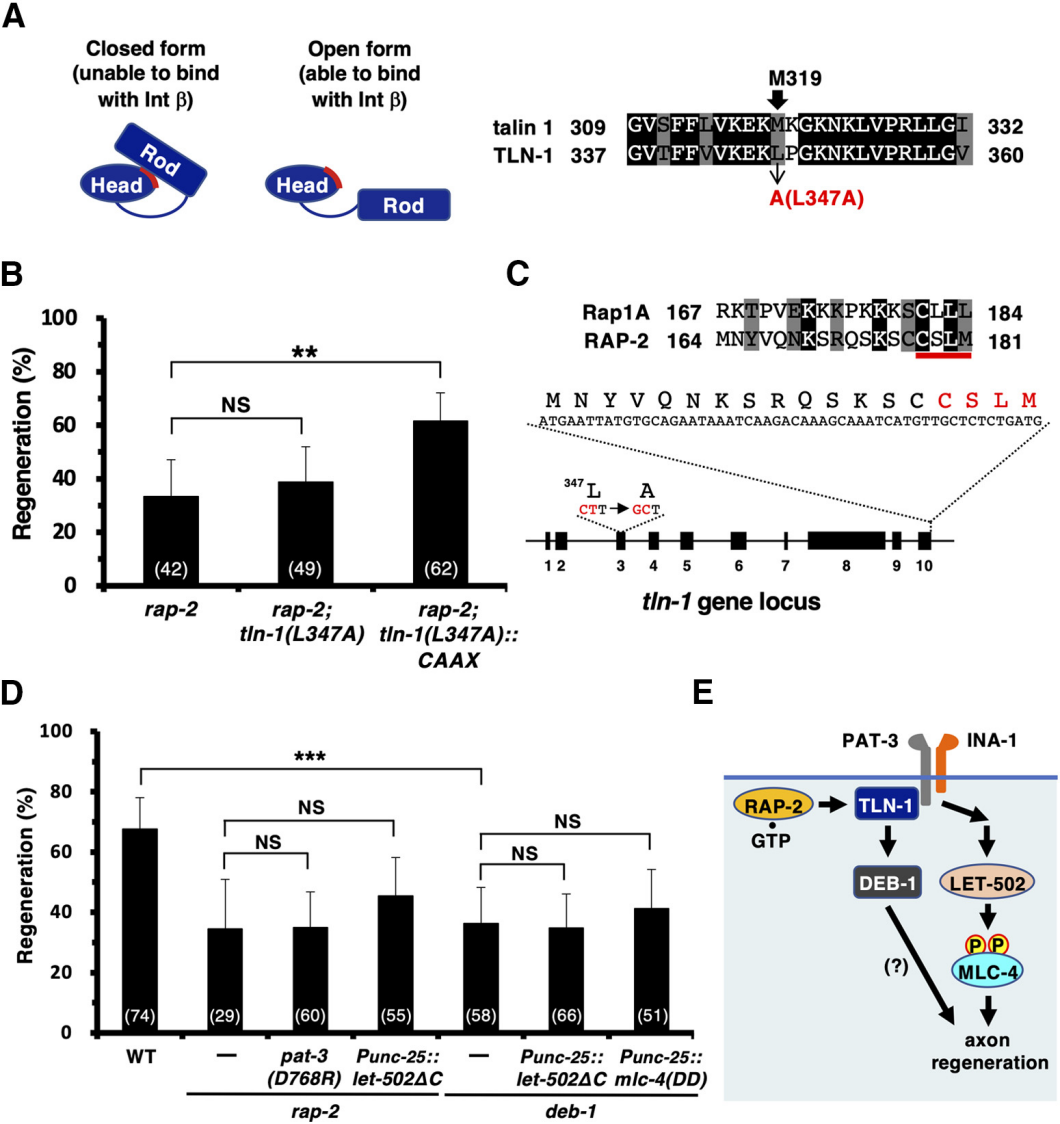
RAP-2 activates TLN-1 in axon regeneration. ***A***, Schematic of the domain organization of talin in the open form (right) and the closed, autoinhibited form (left). Talin is autoinhibited by the interaction of the N-terminal head domain with the C-terminal rod domain, which prevents the interaction of the head domain with the membrane surface and β-integrin cytoplasmic tail. The talin head domain is composed of four subdomains: F0, F1, F2, and F3. Sequence alignment of F3 subdomains between talin 1 and TLN-1 is shown. Identical and similar residues are highlighted with black and gray shading, respectively. The Met-319 site in the talin F3 domain plays a crucial role in interacting with the C-terminal rod domain. The conserved residues Met-319 (in talin 1) and Leu-347 (in TLN-1) are indicated by arrows. ***B***, ***D***, Percentages of axons that initiated regeneration 24 h after laser surgery in the young adult stage. The numbers of axons examined are shown. Error bars indicate 95% confidence intervals. **p < 0.01, ***p < 0.001, as determined by Fisher's exact test. NS, Not significant. ***C***, Introduction of the RAP-2 CAAX box into the *tln-1* locus. Sequence alignment of CAAX boxes (red line) in Rap1 and RAP-2 is shown. Identical and similar residues are highlighted with black and gray shading, respectively. The C-terminal 18 residues of RAP-2 were inserted by CRISPR/Cas9 mutagenesis into the C-terminal site of the *tln-1* locus carrying the *L347A* mutation. ***E***, Partners of TLN-1. TLN-1 interacts with PAT-3 and DEB-1. WT, Wild type.

Rap1 plays a role in activating talin by recruiting it to the plasma membrane, where the autoinhibitory interaction between the talin N-terminal head and C-terminal rod domains is disrupted, which can expose the integrin β-tail binding site in talin (Fig. 4*A*; Gingras et al., 2019). A recent study using nuclear magnetic resonance spectroscopy elucidated the structural basis for talin autoinhibition and revealed that the Met-319 site plays a crucial role in the interaction with the C-terminal rod domain of talin (Goksoy et al., 2008). Therefore, the M319A mutation disrupts the talin autoinhibitory interaction, thereby inducing its constitutively open conformation and activation (Fig. 4*A*). The region around the Met-319 site in mammalian talin is conserved in *C. elegans* TLN-1, with the Leu-347 residue corresponding to Met-319 (Fig. 4*A*). We speculated that the *tln-1(L347A)* mutation would constitutively activate TLN-1; however, the *tln-1(L347A)* mutation failed to suppress the axon regeneration defect observed in *rap-2(gk11)* mutants (Fig. 4*B*, Table 2). Because recruitment of talin to the membrane is required for integrin activation (Goksoy et al., 2008), the ability of the *tln-1(L347A)* mutation to suppress the *rap-2(gk11)* defect in axon regeneration may be dependent on membrane localization of TLN-1(L347A). Rap1 contains a C-terminal CAAX box that plays an important role in its localization to cellular membranes (Hancock, 2003). To investigate the effect of the membrane localization of TLN-1(L347A) on the suppression of the *rap-2(gk11)* phenotype, we used CRISPR/Cas9 mutagenesis to introduce the C-terminal 18 residues of RAP-2, which contain the CAAX box, into the COOH-terminal site at the *tln-1* locus carrying the *L347A* mutation (Fig. 4*C*). We found that *tln-1(L347A)::CAAX* was able to suppress the axon regeneration defect in *rap-2(gk11)* mutants (Fig. 4*B*, Table 2). These results suggest that TLN-1(L347A)::CAAX bypasses the requirement for RAP-2 activity to activate integrin in the axon regeneration pathway.

Next, we tested linking RAP-2 to the PAT-3−LET-502/ROCK−MLC-4 phosphorylation pathway in axon regeneration. We found that the regeneration defect in *rap-2(gk11)* mutants was not suppressed by the constitutively active *pat-3(D768R)* mutation or expression of LET-502ΔC in D neurons (Fig. 4*D*, Table 2). Because RAP-2 regulates axon regeneration by activating TLN-1, TLN-1 may mediate signals through two different pathways to promote axon regeneration. A possible second target for a TLN-1-mediated regeneration pathway might be identified by noting that talin also activates vinculin, an essential linker protein between the actin cytoskeleton and ECM-bound integrins (Izard et al., 2004; Parsons et al., 2010). Loveless et al. (2017) demonstrated that the *C. elegans* vinculin ortholog DEB-1 is associated with TLN-1 (Fig. 4*E*). Therefore, we examined whether DEB-1 participates in axon regeneration and observed that *deb-1(gk329549)* mutants were impaired in axon regeneration (Fig. 4*D*, Table 2). In contrast to *pat-3(gk804163)* mutants, expression of LET-502ΔC or MLC-4(DD) failed to suppress the *deb-1(gk329549)* phenotype (Fig. 4*D*, Table 2). These results suggest that TLN-1 regulates axon regeneration via PAT-3- and DEB-1-mediated pathways. Based on these results, it is possible that the *tln-1(e259)* mutation is defective in activating the PAT-3 pathway but is capable of activating the DEB-1 pathway.

### EPAC-1/Epac activates RAP-2 GTPase to promote axon regeneration

**Figure 5.**
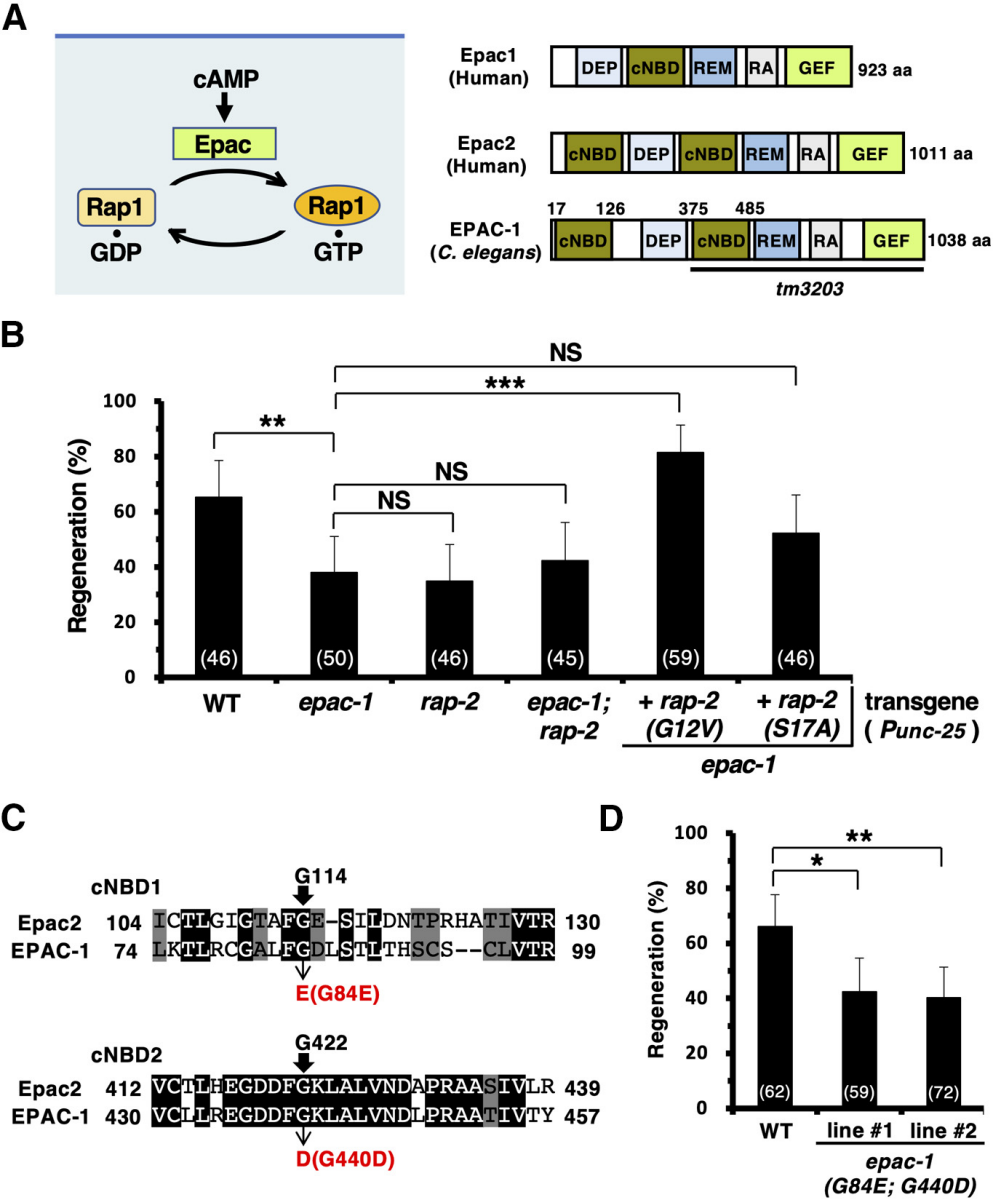
EPAC-1 activates RAP-2 to promote axon regeneration. ***A***, Activation of Rap1 by Epac. Schematic diagrams of EPAC-1 and human Epac1 and Epac2 are shown. DEP, Dishevelled, Egl-10, pleckstrin domain; REM, Ras-exchange motif; RA, Ras-associating domain; GEF, GEF catalytic domain. Bold line underneath denotes the extent of the deleted region in the *tm3203* mutant. ***B***, ***D***, Percentages of axons that initiated regeneration 24 h after laser surgery in the young adult stage. The numbers of axons examined are shown. Error bars indicate 95% confidence intervals. *p < 0.05, **p < 0.01, ***p < 0.001, as determined by Fisher's exact test. NS, Not significant. ***C***, cAMP-binding sites in EPAC-1. Sequence alignment of the first and second cNBDs between Epac2 and EPAC-1 is shown. Identical and similar residues are highlighted with black and gray shading, respectively. The Gly-114 and Gly-422 sites in Epac2 are essential for its cAMP binding activity. The conserved residues, Gly-114 (in Epac2), Gly-84 (in EPAC-1), Gly-422 (in Epac2), and Gly-440 (in EPAC-1) are indicated by arrows. WT, Wild type.

We further evaluated the role of RAP-2 in the axon regeneration pathway. Like other GTPases, Rap exists in inactive GDP-bound and active GTP-bound states. A GEF protein promotes the exchange of GDP for GTP and activates its target GTPase (Boguski and McCormick, 1993). A number of GEFs that mediate the activation of mammalian Rap1 have been identified. An intriguing RapGEF is Epac (exchange protein directly activated by cAMP), because this GEF represents a direct target for cAMP, independent of the classical cAMP target, protein kinase A (PKA; de Rooij et al., 1998; Kawasaki et al., 1998). Indeed, Epac is known to be involved in the control of integrin-mediated cell adhesion (Rangarajan et al., 2003), and cAMP also participates in axon regeneration (Bhatt et al., 2004; Pearse et al., 2004; Ghosh-Roy et al., 2010). On the basis of these results, we hypothesized that cAMP-activated Epac is responsible for Rap activation, which leads to talin-mediated integrin activation in axon regeneration (Fig. 5*A*). The *epac-1* gene encodes the *C. elegans* homolog of Epac (Fig. 5*A*; Tada et al., 2012). We therefore examined whether *epac-1* is required for axon regeneration, and we found that *epac-1(tm3203)* mutants exhibited a phenotype defective in axon regeneration (Fig. 5*B*, Table 2). We then analyzed the genetic interaction of *epac-1* with *rap-2*. We found that *epac-1(tm3203)*; *rap-2(gk11)* double mutants were almost as defective in axon regeneration as *rap-2(gk11)* single mutants (Fig. 5*B*, Table 2). These results suggest that *rap-2* and *epac-1* act on the same axis controlling axon regeneration. Furthermore, we found that the expression of constitutively active RAP-2(G12V), but not inactive RAP-2(S17A) (Fig. 3*B*), from the *unc-25* promoter in D-type motor neurons could suppress the *epac-1* phenotype (Fig. 5*B*, Table 2), suggesting that EPAC-1 is a GEF for RAP-2 in axon regeneration.

Next, we examined whether cAMP binding is important for EPAC-1 to promote axon regeneration. Epac contains a cyclic nucleotide binding domain (cNBD), which is fused directly to the GEF domain as a single polypeptide chain (Fig. 5*A*; Bos, 2003). cNBD folds on the GEF domain and prevents its interaction with downstream effectors. Binding of cAMP to Epac results in a conformational change that allows its GEF domain to interact with Rap, leading to Rap activation and subsequent downstream effects. *C. elegans* EPAC-1 contains two cNBDs, the first at the N terminus and the second in the middle region (Fig. 5*A*; Bos, 2003). The human Epac2(G114E; G422D) mutant, wherein both Gly-114 and Gly-422 were replaced with glutamic acid and aspartic acid residues, respectively, has been shown to be defective in cAMP binding (Fig. 5*C*; Ozaki et al., 2000). *C. elegans* EPAC-1 possesses conserved sites Gly-84 and Gly-440 in the first and second cNBDs, corresponding to Gly-114 and Gly-422 in Epac2, respectively (Fig. 5*C*). To determine whether the cAMP binding activity of EPAC-1 is important for axon regeneration, we generated *epac-1(G84E; G440D)* knock-in mutants using the CRISPR/Cas9 technique. We found that *epac-1(G84E; G440D)* mutants had significantly reduced axonal regeneration (Fig. 5*D*, Table 2), indicating that EPAC-1 is required for axon regeneration in a manner dependent on its cAMP binding activity. Altogether, these results suggest that axon injury leads to increased cAMP levels, which, in turn, activate the EPAC-1−RAP-2−TLN-1-mediated integrin inside-out activation signaling pathway to promote axon regeneration.

### Integrin activates the RhoA signaling pathway via src and RhoGEF in axon regeneration

**Figure 6.**
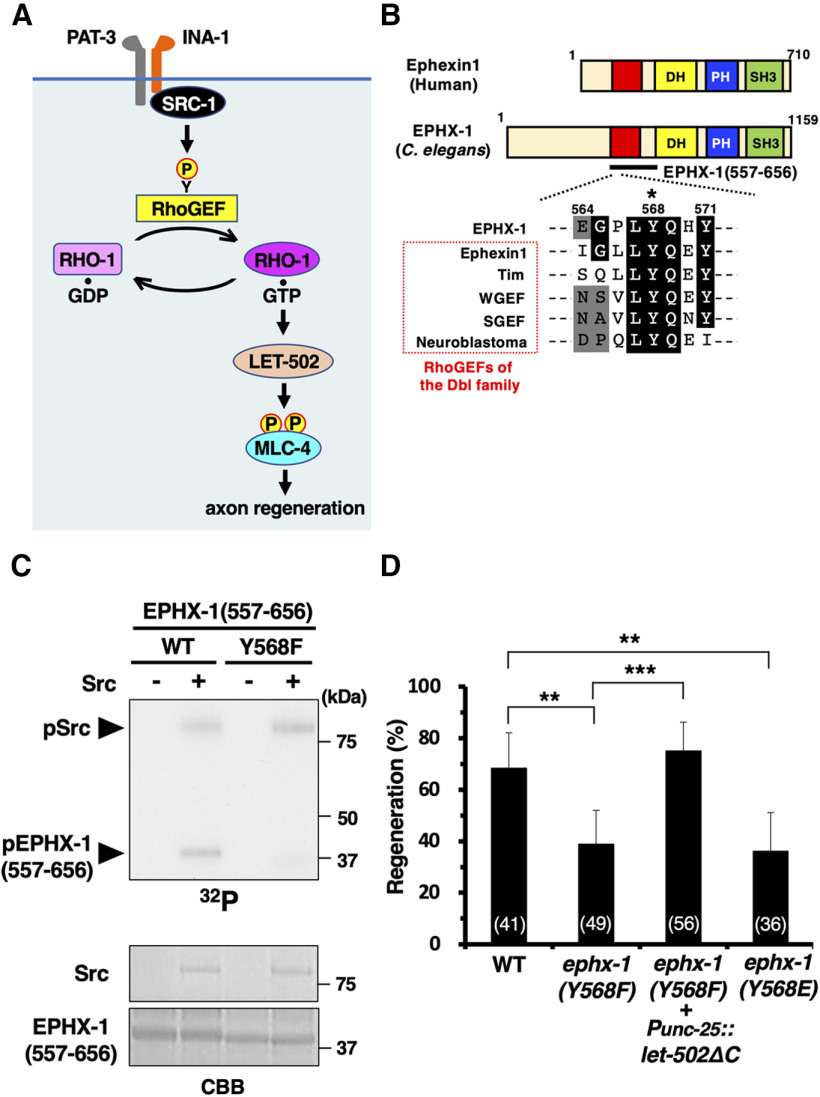
Src phosphorylation of EPHX-1 RhoGEF is essential for axon regeneration. ***A***, Activation of the RHO-1−LET-502 pathway by SRC-1 and RhoGEF in axon regeneration. SRC-1 activates RhoGEF by phosphorylating a specific tyrosine residue, which, in turn, leads to the activation of RHO-1. GTP-bound active RHO-1 activates LET-502, resulting in MLC-4 phosphorylation and promoting axon regeneration. ***B***, Structure of EPHX-1. Schematic diagrams of EPHX-1 and mammalian ephexin1 are shown. DH domain, yellow; PH domain, blue; SH3 domain, green. Conserved autoinhibitory regions of EPHX-1 and RhoGEFs of the Dbl family are shown in red and highlighted in the multiple-sequence alignment. Identical and similar residues are highlighted with black and gray shading, respectively. Tyr-568 of EPHX-1 is indicated by an asterisk. The EPHX-1(557–656) region is shown (underlined). ***C***, Src phosphorylation of EPHX-1. *In vitro* phosphorylation of EPHX-1(557–656) by Src is shown. COS-7 cells were transfected with GFP-EPHX-1(557–656) or GFP-EPHX-1(557–656; Y568F), and cell lysates were immunoprecipitated with an anti-GFP antibody. Immunoprecipitates were incubated with active recombinant Src in the presence of [γ-^32^P]ATP for 20 min at 30°C. Autophosphorylated Src and phosphorylated GFP-EPHX-1(557–656) were resolved by SDS-PAGE (top panel, ^32^P). Protein input was confirmed by Coomassie Brilliant Blue (CBB) staining. ***D***, Percentages of axons that initiated regeneration 24 h after laser surgery in the young adult stage. The numbers of axons examined are shown. Error bars indicate 95% confidence intervals. **p < 0.01, ***p < 0.001, as determined by Fisher's exact test. WT, Wild type.

How does the inside-out activation of integrin regulate the RhoA signaling pathway in axon regeneration? Since RhoA activation depends on RhoGEF activity that catalyzes the GDP–GTP exchange reaction (Cerione and Zheng, 1996), a GEF for the RHO-1 GTPase should function downstream of the integrin signaling pathway in regulating axon regeneration (Fig. 6*A*). One potential mediator between integrin and RhoA activation is the nonreceptor tyrosine kinase Src. Mammalian Src family kinases can interact directly or indirectly with integrins to activate RhoGEFs of the Dbl family, such as ephexin, through tyrosine phosphorylation (Arias-Salgado et al., 2003; Sahin et al., 2005; Huveneers and Danen, 2009). *C. elegans* SRC-1/Src interacts directly with INA-1/integrin α and mediates integrin signaling in phagocytic cells (Hsu and Wu, 2010). Furthermore, we have previously demonstrated that SRC-1 and INA-1 are required for axon regeneration (Pastuhov et al., 2016). On the basis of these results, we hypothesized that SRC-1 transduces integrin signaling to activate RHO-1 by phosphorylating, thereby activating ephexin-like RhoGEF (Fig. 6*A*). The *C. elegans* genome contains EPHX-1, a homolog of mammalian ephexin, that belongs to the Dbl family (Fig. 6*B*). We therefore investigated the relationship between SRC-1 and EPHX-1 in the axon regeneration pathway. Interestingly, the Src phosphorylation site in ephexin is also conserved in EPHX-1, corresponding to the Tyr-568 residue (Fig. 6*B*). To test whether EPHX-1 Tyr-568 is phosphorylated by Src, *in vitro* kinase assays were performed. Since the full-length recombinant EPHX-1 protein obtained from *Escherichia coli* was insoluble, a shorter EPHX-1 fragment containing the Tyr-568 site (amino acids 557−656; Fig. 6*B*) was immunopurified from COS-7 cells expressing GFP-EPHX-1(557−656). We incubated active mammalian Src with EPHX-1(557 − 656) *in vitro* and observed the phosphorylation of EPHX-1(557− 656; Fig. 6*C*). Mutating Tyr-568 to phenylalanine in EPHX-1(557−656) substantially reduced its *in vitro* phosphorylation by Src (Fig. 6*C*). These results suggest that EPHX-1 Tyr-568 represents a substrate for direct phosphorylation by Src.

We next addressed the biological importance of EPHX-1 Tyr-568 phosphorylation in axon regeneration. We generated the phosphorylation-defective *ephx-1(Y568F)* mutation at the *ephx-1* locus by the CRISPR/Cas9 mutagenesis. We found that *ephx-1(Y568F)* mutants were defective in axon regeneration (Fig. 6*D*, Table 2). Furthermore, the expression of a constitutively active LET-502ΔC from the *unc-25* promoter could suppress the *ephx-1(Y568F)* mutant phenotype (Fig. 6*D*, Table 2). To determine whether acidification of the Tyr-568 site would cause constitutive activation, we constructed the phosphomimetic *ephx-1(Y568E)* mutation at the *ephx-1* locus. However, the *ephx-1(Y568E)* mutation lost its ability to promote regeneration (Fig. 6*D*, Table 2), suggesting that the acidic amino acid could not substitute for tyrosine phosphorylation (Hoppmann et al., 2017). Therefore, Tyr-568 phosphorylation of EPHX-1 is necessary for its function as a GEF for RHO-1.

### EPHX-1 N-terminal domain inhibits interaction with RHO-1

**Figure 7.**
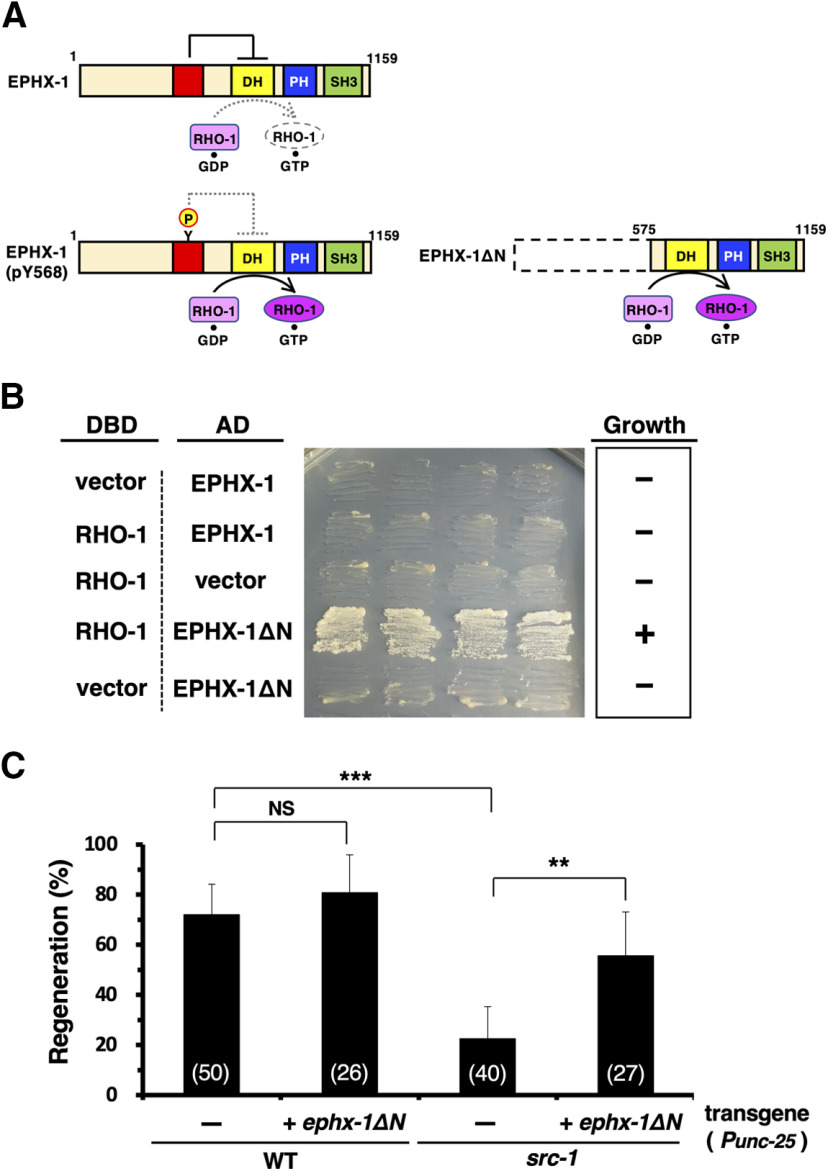
EPHX-1 N-terminal domain inhibits interaction with RHO-1. ***A***, The autoinhibition model for EPHX-1. The autoinhibitory helix (red) packs against a conserved pocket on the DH domain (yellow), which inhibits the interaction between RHO-1 and the DH domain. The tyrosine phosphorylation of the autoinhibitory helix causes its dissociation from the DH domain, resulting in the interaction with RHO-1. EPHX-1ΔN lacking amino acids 1–574 can constitutively associate with RHO-1. ***B***, Yeast two-hybrid assays for the interactions of RHO-1 with EPHX-1 and EPHX-1ΔN. The reporter strain PJ69-4A was cotransformed with expression vectors encoding GAL4 DBD-RHO-1, GAL4 AD-EPHX-1, and GAL4 AD-EPHX-1ΔN, as indicated. Yeasts carrying the indicated plasmids were grown on selective plates lacking histidine and containing 10 mm 5-aminotriazole for 4 d. ***C***, Percentages of axons that initiated regeneration 24 h after laser surgery in the young adult stage. The numbers of axons examined are shown. Error bars indicate 95% confidence intervals. **p < 0.01, ***p < 0.001, as determined by Fisher's exact test. WT, Wild type.

RhoGEFs of the Dbl family possess tandem Dbl homology (DH), pleckstrin homology (PH), and SH3 domains (Fig. 7*A*; Schmidt and Hall, 2002). Many Dbl family proteins are autoinhibited by direct binding of a putative helix N terminal to the DH domain, which sterically hinders Rho GTPases and prevents activation (Fig. 7*A*; Aghazadeh et al., 2000; Yohe et al., 2008). This autoinhibition is relieved by the Src phosphorylation of the tyrosine residue in the autoinhibitory helix, which disrupts the interaction with the DH domain. Because Src phosphorylation of EPHX-1 Tyr-568 was required for its function in axon regeneration (Fig. 6*D*, Table 2), we expected that the deletion of the N terminus containing Tyr-568 (amino acids 1–574; EPHX-1ΔN; Fig. 7*A*) would generate a hyperactive EPHX-1. To demonstrate the influence of the EPHX-1 N-terminal domain in regulating its GEF activity, we examined the ability of EPHX-1ΔN to interact with RHO-1 using a yeast two-hybrid system. We found that EPHX-1ΔN formed a complex with RHO-1, whereas full-length EPHX-1 did not associate with RHO-1 (Fig. 7*B*). These results suggest that the access of RHO-1 to its binding DH domain is restricted by the N terminus of EPHX-1.

The increased activity of EPHX-1ΔN was also apparent in testing the epistatic relation between *src-1* and *ephx-1* in the regulation of axon regeneration. The expression of *ephx-1*Δ*N* from the *unc-25* promoter could suppress the regeneration defect of *src-1(cj293)* mutants (Fig. 7*C*, Table 2). Based on these findings, we propose that the EPHX-1 N-terminal domain has an autoinhibitory function, which is released by SRC-1 phosphorylation of Tyr-568, resulting in the activation of RHO-1. Thus, SRC-1 functions upstream of EPHX-1 in the RHO-1–LET-502-mediated signaling pathway to regulate axon regeneration.

## Discussion

Integrin has previously been implicated in axonal regeneration in both the peripheral nervous system (PNS) and CNS (Eva and Fawcett, 2014). Successful regeneration in the PNS following injury has been demonstrated correlating with the upregulation of specific integrin subunits, including α4, α5, α6, α7, and β1 (Vogelezang et al., 2001; Ekström et al., 2003; Wallquist et al., 2004; Gardiner et al., 2007), suggesting the importance of different types of integrins in neuronal regeneration. In fact, the axonal regeneration of the facial nerve in mice defective in integrin α7 is severely impaired (Werner et al., 2000). In the case of CNS injury, upregulation of inhibitory molecules such as chondroitin sulfate proteoglycans and Nogo-A in a pathologic environment can result in integrin inactivation (Hu and Strittmatter, 2008; Tan et al., 2011), whereas induced activation can allow axons to overcome those inhibitory effects on regeneration (Tan et al., 2011). Thus, it is clear that the activation state of integrin is an important factor in achieving significant regenerative growth. However, the molecular mechanisms by which integrin contributes to axonal regeneration have been poorly described. Here, we show that the nonreceptor tyrosine kinase Src and its target ephexin mediate integrin signaling to promote axon regeneration through the RhoA–ROCK–MLC phosphorylation pathway in *C. elegans*. Our findings thus provide a valuable molecular insight into the integrin-regulated repair of damaged neurons.

**Figure 8.**
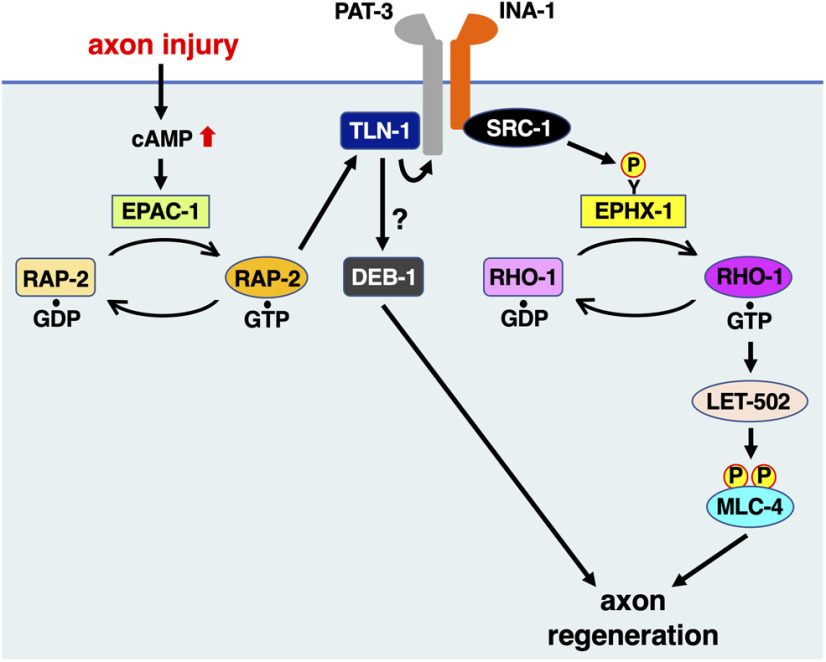
Schematic model for the regulation of axon regeneration by the integrin signaling network. In response to axon injury, cAMP levels are elevated, resulting in the activation of EPAC-1, which, in turn, activates RAP-2. GTP-bound RAP-2 can interact with and activate TLN-1, which then activates integrin and possibly DEB-1 as well. Next, integrin activation directs the GDP–GTP exchange activity of EPHX-1 toward RHO-1 via SRC-1-mediated phosphorylation of EPHX-1 on Tyr-568. Finally, GTP-bound RHO-1 activates LET-502, leading to MLC-4 phosphorylation, which promotes axon regeneration.

In this study, we demonstrate that integrin activation is essential for creating a functional transmembrane receptor that can induce downstream cellular effects in axon regeneration. A key step in the inside-out activation of integrin signaling is the binding of talin to the cytoplasmic domain of the β-subunit (Kim et al., 2011). When the receptor is in the bent, inactivated state, a salt bridge is formed between the KLLxIIHD motif on the β-subunit and GFFKR motif on the α-subunit. When talin binds to the cytoplasmic tail of the β-subunit, the transmembrane domains of α- and β-integrins separate, resulting in a conformational change that modulates downstream signaling. The mutated form of the integrin β-subunit *D759R* (numbered using the β1 sequence) leads to constitutive activation of integrin by disrupting the salt bridge between the α- and β-subunits (Laursen et al., 2011). The conserved sequences GFFKR and KLLtVLHD are also present in INA-1/integrin α and PAT-3/integrin β, respectively. This raises the possibility that talin-mediated inside-out activation of integrin may be involved in the regulation of axon regeneration. Indeed, we found that the *pat-3*, *ina-1*, and *tln-1* mutants are defective in axon regeneration and that the *pat-3(D768R)* mutation, corresponding to the mammalian integrin β1(D759R) mutation, can suppress the *tln-1* mutant phenotype, suggesting that the *pat-3(D768R)* mutation is constitutively active. These results are consistent with the possibility that the TLN-1/talin-mediated activation of PAT-3/integrin β promotes axon regeneration (Fig. 8).

How is TLN-1 activated in response to axonal injury? Mammalian talin exists in the cytoplasm in a closed, autoinhibited conformation, in which intramolecular interactions between the N-terminal and C-terminal domains prevent integrin binding (Goksoy et al., 2008). Upon stimulation, talin is efficiently recruited to the cell membrane and released from its autoinhibitory conformation to trigger integrin activation (Song et al., 2012). It has been suggested that the Rap1 GTPase plays a critical role in activating talin by recruiting it to the membrane, relieving the autoinhibitory interactions (Zhu et al., 2017). In addition, Rap1 is activated by GEF Epac1, which is a target of cAMP (de Rooij et al., 1998; Kawasaki et al., 1998). The present study provides compelling evidence that the EPAC-1/Epac–RAP-2 GTPase signaling pathway activates TLN-1/talin-mediated integrin activation in axon regeneration (Fig. 8). In the absence of cAMP, mammalian Epac has been reported to exist in an inactive conformation such that access of Rap1 to the GEF domain of Epac is occluded by an intramolecular interaction between the cAMP-binding domain and catalytic region (Rehmann et al., 2003, 2006). Upon cAMP accumulation, nucleotide binding promotes conformational change in Epac that uncouples the cAMP-binding domain from the catalytic region, enabling Rap1 activation. Thus, the Epac–Rap1 pathway represents a PKA-independent cAMP signaling cascade. The secondary messenger cAMP is involved in axonal regeneration through the activation of PKA (Gao et al., 2004). However, an additional cAMP-dependent mechanism involves EPAC-1, which also responds to physiological changes in cAMP concentration, promoting the activation of the RAP-2 GTPase.

Since the artificial localization of TLN-1 to the membrane can suppress the defect in axon regeneration caused by the *rap-2* mutation, the main role of RAP-2 in axon regeneration is to recruit TLN-1 to the membrane. However, the constitutively active *pat-3(D768R)* mutation or LET-502ΔC expression is unable to suppress the *rap-2* defect. Thus, the PAT-3–LET-502 pathway does not appear to be simply downstream of RAP-2 signaling, but another pathway is also likely to be important for axon regeneration. A possible factor in this other pathway might be vinculin, an essential linker protein between the actin cytoskeleton and integrins bound to the ECM. Upon activation by talin, vinculin binds to both integrin-bound talin and actin filaments, allowing cells to transmit the force generated by actomyosin to the ECM via the vinculin–talin–integrin complex (Zamir and Geiger, 2001; Izard et al., 2004). In fact, we find that DEB-1/vinculin is involved in axon regeneration. In contrast to *pat-3* mutants, the expression of LET-502ΔC does not suppress the *deb-1* mutant defect in axon regeneration. Based on these results, we hypothesize that TLN-1 regulates axon regeneration through PAT-3- and DEB-1-mediated pathways (Fig. 8).

What are mediators of the transduction of integrin signals leading to RHO-1/RhoA GTPase activation? In general, Rho GTPases are activated by RhoGEFs, so we expected that a RhoGEF would be involved in regulating the axon regeneration pathway between integrin and RHO-1. Mammalian integrin α_4_–β_1_ acts via the α_4_ cytoplasmic domain to activate downstream Src, which, in turn, activates the GEF complex that acts on Rac GTPase to promote cell motility (Hsia et al., 2005). These results, combined with our findings, define a conserved integrin signaling pathway, in which activation of SRC-1 by integrin provides a link to RhoGEF-mediated RHO-1 activation during axon regeneration in *C. elegans*. We demonstrate that RhoGEF EPHX-1 acts downstream of SRC-1 in the integrin–RHO-1 signaling pathway (Fig. 8). EPHX-1 is a homolog of the mammalian RhoGEF ephexin, which belongs to the Dbl family of proteins. The Dbl family GEFs share the structural motif of the central DH catalytic domain in tandem with the regulatory PH domain. The N-terminal half of Dbl RhoGEFs has been proven to be characterized by a negative regulatory element for the DH–PH functional module (Aghazadeh et al., 2000; Yohe et al., 2007, 2008; Tanegashima et al., 2008). This intramolecular interaction hinders the Rho GTPase access to the DH domain needed to catalyze the guanine nucleotide exchange. Mammalian ephexin is phosphorylated at a tyrosine residue in an N-terminal motif and has considerable sequence identity with the autoinhibitory helix described for the members of the Dbl family (Sahin et al., 2005; Yohe et al., 2008). Upon the phosphorylation of this tyrosine residue in the N-terminal region by Src, Dbl RhoGEFs open to yield an active conformation with an exposed DH domain, relieving this autoinhibition. Indeed, the autoinhibitory helix is conserved between EPHX-1 and Dbl family members, and we observed a similar negative regulation of EPHX-1 by its N-terminal domain, as shown by the hyperactivity of the N-terminal truncation mutant. Src phosphorylation of Tyr-568 in the N-terminal domain of EPHX-1 suggests that this tyrosine phosphorylation induces EPHX-1 activation.

In the mammalian Dbl family neuronal guanine exchange factor (Ngef), Tyr-179 is phosphorylated by Src, and substitution of Tyr-179 with glutamic acid (Y179E) causes constitutive activation of Ngef GEF activity (Yohe et al., 2008). In contrast, the Y568E mutation in EPHX-1 is loss of function, which indicates that tyrosine phosphorylation is important for the GEF activity of EPHX-1. The nucleotide exchange activity of the Dbl family RhoGEFs is autoinhibited by an additional intramolecular interaction between the N- and C-terminal regions (Yohe et al., 2008). Binding of another protein to the N-terminal region would activate the GEF activity by disrupting this intramolecular interaction. One potential binding partner for EPHX-1 might be SRC-1 itself. Here, after SRC-1 phosphorylates EPHX-1 at Tyr-568, the SH2 domain of SRC-1 associates with pTyr-568 in EPHX-1, resulting in the activation of EPHX-1 GEF activity by relieving the autoinhibitory intramolecular interaction. Because SRC-1 associates with INA-1 (Hsu and Wu, 2010), this possibility also suggests that SRC-1 acts on EPHX-1 in the vicinity of the integrin receptor. If so, the interaction of TLN-1 with PAT-3 could induce the binding of SRC-1 to INA-1. Therefore, SRC-1 may not only promote GEF activity of EPHX-1, but also determine its subcellular localization.

We have previously shown that the integrin pathway also promotes axon regeneration through activation of the JNK cascade (Pastuhov et al., 2016; Hisamoto et al., 2019). In this pathway, SRC-1 provides a link between INA-1 and the GEF complex CED-2/CrkII, CED-5/DOCK180, and CED-12/ELMO, which activates CED-10/Rac GTPase. GTP-bound CED-10 interacts with and activates the Ste20-related protein kinase MAX-2, which phosphorylates and activates MLK-1 MAPKKK (Pastuhov et al., 2016). In this JNK pathway, integrin is activated by externalized phosphatidylserine generated by axon severing (Hisamoto et al., 2018). Therefore, SRC-1 activates two separate signaling pathways via RhoGEFs, one that leads to activation of the Rac–JNK pathway, and a second that leads to activation of the RhoA–ROCK pathway that is linked to SRC-1 by TLN-1. Moreover, we have recently identified the *C. elegans* tensin protein TNS-1 as an adaptor protein for the SVH-2 Met-like receptor–JNK signaling pathway (Hisamoto et al., 2019). TNS-1 brings PAT-3 in close proximity to SVH-2 by associating with both proteins. Hence, it is likely that TNS-1 links SRC-1 to integrin–JNK signaling.
